# Supplementary material for: Misconceptions of Antibiotics as a Potential Explanation for Their Misuse. A Survey of the General Public in a Rural and Urban Community in Sri Lanka
Source: Antibiotics (Basel). 2022 Mar 27;11(4):454. doi: 10.3390/antibiotics11040454 (PMC9024968; doi:10.3390/antibiotics11040454)
Supplement: Supplementary file 1 [file antibiotics-11-00454-s001.zip › antibiotics-1617744-supplementary.pdf]

0

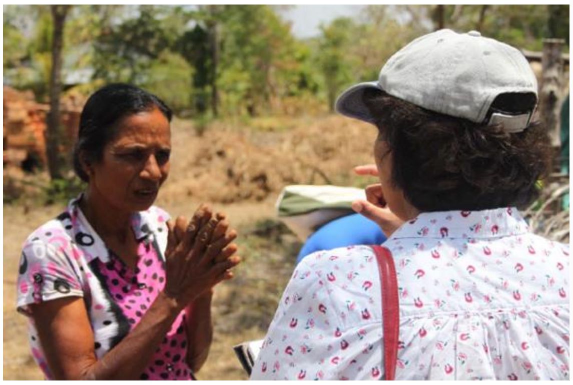

Knowledge and understanding of antimicrobial use and antimicrobial resistance

Thank you for agreeing to complete this questionnaire.

I am a researcher at the Department of Veterinary Public Health and Pharmacology at the University of Peradeniya. This survey is part of an international, collaborative project undertaken by the University of Peradeniya and the Royal Veterinary College, University of London.

The aim of this survey is to explore how much you know about antibiotics and antibiotic resistance. Your participation will enable us to understand more clearly the general public's understanding and perceptions on this topic.

The survey forms part of a larger study investigating the potential role of wildlife in antimicrobial resistance and ecosystem contamination in Sri Lanka. Information collected from this survey will be used for this project only and will not be shared with anyone for any other purpose; your identity will remain anonymous.

If you would like further information about the study, please do not hesitate to contact me: yadeegunasekara@gmail.com

Thank you

Please click or mark the boxes below with your answers.

Nature Citizen

Your thoughts on antibiotics

1. How would you rate your knowledge about the medicine group called antibiotics?	Very good	☐	Good	☐
	Poor	☐	Very poor	☐
2. From this list, please identify which medicines you think are antibiotics (Please select only 5 answers):	Paracetamol	☐	Penicillin	☐
	Ampicillin	☐	Folic acid	☐
	Piriton	☐	Tetracycline	☐
	Amoxycillin	☐	Aspirin	☐
	Streptomycin	☐	Panadol	☐
3. List any other antibiotics that you know:				

Personal antibiotic usage

4. Have you ever taken antibiotics?	Yes	☐	No	☐
	I cannot remember	☐	Prefer not to answer	☐
5. If you have taken antibiotics, where did you obtain them? (Please tick all that apply)	From a doctor	☐	From a family friend	☐
	From a pharmacist	☐	Prefer not to answer	☐
6. When did you last take antibiotics?	Within last month?	☐	Within past 6 months?	☐
	Within last year?	☐	More than one year ago?	☐
	I cannot remember	☐	Prefer not to answer	☐
7. On the last occasion you took antibiotics, did you receive advice from a doctor on how to take them? (e.g. how many days, time etc.)	Yes	☐	No	☐
	I cannot remember	☐	Prefer not to answer	☐
8. On the last occasion you took antibiotics, did you receive advice from a pharmacist on how to take them? (e.g. how many days, time etc.)	Yes	☐	No	☐
	I cannot remember	☐	Prefer not to answer	☐

Royal Veterinary College University of London

CEJ Centre for Environmental Justice

University of Peradeniya

9. How do you normally decide on the appropriate period for taking antibiotics? (Please select only ONE answer).	Period indicated by your doctor or pharmacist	☐
	Period indicated by a family member or friend	☐
	Antibiotics should be used until symptoms disappear	☐
	Antibiotics should be used until I get relief from symptoms	☐

10. Whose advice do you take on using and purchasing antibiotics? (You may select more than one answer)	Doctor	☐	Pharmacist	☐
	Friends or family	☐	I purchase antibiotics without any advice	☐
			Prefer not to answer	☐

**11.** Indicate whether the following statements are true or false:	**True**	**False**
It is acceptable to use antibiotics that were given to another person if the antibiotics are used to treat the same symptoms or illness	☐	☐
It is acceptable to buy the same antibiotics, without consulting a doctor or pharmacist, if you are sick and they helped you to fight the same symptoms in the past	☐	☐
It is good to keep leftover antibiotics at home in case of future need	☐	☐
A prescription from a doctor is needed to purchase antibiotics	☐	☐
A pharmacist is capable of prescribing antibiotics	☐	☐
It is acceptable to buy antibiotics for animals without advice from a veterinary doctor	☐	☐

12. Please indicate which of the following can be effectively treated with antibiotics:	Bacteria	☐	Virus	☐
	Bacteria and virus	☐	Do not know	☐

13. Do you think people can be allergic to antibiotics?	Yes	☐	No	☐

14. Which of the following diseases or disorders do you think can be treated with antibiotics?

HIV/AIDS	☐	Diarrhoea	☐	Cold	☐
Sore throat	☐	Fever	☐	Malaria	☐
Skin rash	☐	Flu	☐	Wound	☐
Body aches	☐	Headaches	☐	I do not know	☐

Nature Citizen

Your thoughts on antibiotic resistance

15. Do you think that there are any health dangers to yourself or other people associated with taking antibiotics?

Yes	No
☐	☐

16. How would you rate your knowledge about the term antibiotic resistance?

(If you rate your understanding of antibiotic resistance as poor or very poor, do not worry, please continue, and try to answer the questions below)

Very good	☐	Good	☐
Poor	☐	Very poor	☐

17. Which sources do you think would be useful in acquiring knowledge about antibiotic resistance? (You may select more than one answer)

Doctor	☐	Pharmacist	☐	Family member or friend	☐
Newspaper	☐	Broadcast	☐	Internet	☐
None	☐	Other	☐		

18. Indicate whether the following statements are true or false:	**True**	**False**
Antibiotic resistance occurs when your body becomes resistant to antibiotics and they no longer work	☐	☐
Many infections are becoming increasingly resistant to treatment by antibiotics	☐	☐
If bacteria are resistant to an antibiotic, it can be difficult or impossible or treat the infection	☐	☐
Antibiotic resistance is an issue that could affect my family and me	☐	☐
Antibiotic resistance is an issue in other countries but not in Sri Lanka	☐	☐
Antibiotic resistance is only a problem for people who take antibiotics regularly	☐	☐
Antibiotic resistance could make medical procedures such as surgery and organ transplant very risky	☐	☐
Bacteria that are resistant to antibiotics can spread from person to person	☐	☐

19. In your opinion, are there enough rules and regulations in Sri Lanka to minimize the occurrence and development of antibiotic resistance?	Yes ☐	No ☐	I do not know ☐

20. Do you agree or disagree with the following statements?

	**Agree strongly**	**Agree slightly**	**Neither agree nor disagree**	**Disagree slightly**	**Disagree strongly**
Antibiotic resistance is one of the biggest problems the world faces today	☐	☐	☐	☐	☐
I am worried about the impact that antibacterial resistance will have on my life and that of my family	☐	☐	☐	☐	☐
I am not at risk of developing antibacterial resistance to infection, if I take my antibiotics correctly	☐	☐	☐	☐	☐
People should use antibiotics only when they are prescribed by a doctor	☐	☐	☐	☐	☐
People should not use leftover antibiotics from a previous treatment to cure other diseases	☐	☐	☐	☐	☐
People like me can do little to combat resistance to antibiotics	☐	☐	☐	☐	☐
Everyone needs to use antibiotics responsibly	☐	☐	☐	☐	☐

Demographic characteristics

21. Gender:

Female	☐	Male	☐	Other	☐	Prefer not to answer	☐

22. Age:

Under 18	☐	18 – 40	☐	41 – 60	☐	More than 60	☐
Prefer not to answer	☐						

23. Marital status:

Married	☐	Single	☐	Divorced / Widowed	☐	Prefer not to answer	☐

24. Please indicate which racial group you belong to:

Sinhalese	☐	Tamil	☐	Muslim	☐	Other	☐
Prefer not to answer	☐						

25. Please indicate the highest level of education achieved:

Primary school	☐	Up to O/L	☐	University	☐	Diploma / trade / technical training	☐
Never been to school	☐	Prefer not to answer	☐	Upto A/L 6			

TO BE COMPLETED BY RESEARCH ASSISTANT

Survey completed with help of research assistant?	Yes	☐	No	☐
Date:	Click or tap to enter a date.			

Royal Veterinary College University of London

CEJ

University of Peradeniya
